# Predicting transcription factor binding using ensemble random forest models

**DOI:** 10.12688/f1000research.16200.2

**Published:** 2019-09-02

**Authors:** Fatemeh Behjati Ardakani, Florian Schmidt, Marcel H. Schulz

**Affiliations:** 1 High throughput Genomics and Systems Biology, Cluster of Excellence on Multimodel Computing and Interaction, Saarland University, Saarbruecken,, Saarland, 66123, Germany; 2 Computational Biology and Applied Algorithmics, Max Planck Institute for Informatics, Saarbruecken, Saarland, 66123, Germany; 3 Graduate School of computer science, Saarland University, Saarbruecken, Saarland, 66123, Germany; 4 Computational Systems Biology, Genome Institute of Singapore, Singapore, Singapore; 5 Institute for Cardiovasular Regeneration, Goethe University Frankfurt Am Main, Frankfurt Am Main, Hessen, 60590, Germany

**Keywords:** ENCODE-DREAM in vivo Transcription Factor binding site prediction challenge, Transcription Factors, Chromatin accessibility, Ensemble learning, Indirect-binding, TF-complexes, DNase1-seq

## Abstract

**Background**: Understanding the location and cell-type specific binding of Transcription Factors (TFs) is important in the study of gene regulation. Computational prediction of TF binding sites is challenging, because TFs often bind only to short DNA motifs and cell-type specific co-factors may work together with the same TF to determine binding. Here, we consider the problem of learning a general model for the prediction of TF binding using DNase1-seq data and TF motif description in form of position specific energy matrices (PSEMs).

**Methods:** We use TF ChIP-seq data as a gold-standard for model training and evaluation. Our contribution is a novel ensemble learning approach using random forest classifiers. In the context of the *ENCODE-DREAM in vivo TF binding site prediction challenge* we consider different learning setups.

**Results:** Our results indicate that the ensemble learning approach is able to better generalize across tissues and cell-types compared to individual tissue-specific classifiers or a classifier built based upon data aggregated across tissues. Furthermore, we show that incorporating DNase1-seq peaks is essential to reduce the false positive rate of TF binding predictions compared to considering the raw DNase1 signal.

**Conclusions:** Analysis of important features reveals that the models preferentially select motifs of other TFs that are close interaction partners in existing protein protein-interaction networks. Code generated in the scope of this project is available on GitHub: https://github.com/SchulzLab/TFAnalysis (DOI: 10.5281/zenodo.1409697).

## Introduction

Transcription Factors (TFs) are key players of transcriptional regulation. They are indispensable to maintain and establish cellular identity and are involved in several diseases ^1^. TFs bind to the DNA at distinct positions, mostly in accessible chromatin regions ^2^, and regulate transcription by recruiting additional proteins. The TFs can alter chromatin organization or, for example, recruit an RNA polymerase to initiate transcription ^1^. Hence, to understand the function of TFs it is vital to identify the genomic location of TF binding sites (TFBS). As TFs regulate distinct genes in distinct tissues, these binding sites are tissue-specific ^2^.

Nowadays, the most prevalent and widely used method to experimentally determine TFBS is through ChIP-seq experiments, which can be used to generate genome-wide, tissue-specific maps of *in-vivo* TF binding. However, ChIP-seq experiments are expensive, experimentally challenging, and require an antibody for the target TF. In this work, target TF refers to the TF of interest, i.e. the TF whose binding sites should be determined. To overcome these limitations, a number of computational methods have been developed to pinpoint TFBS. Most of these methods are based on position weight matrices (PWMs) describing the sequence preference of TFs ^3, 4^. PWMs indicate, for each position of a TF binding motif independently, how likely the individual nucleotides are to occur at a specified position. Unfortunately, screening the entire genome using a PWM results in too many false positive predictions. Therefore, numerous methods have been proposed to reduce the prediction error by combining PWMs with epigenetics data, such as DNase1-seq, ATAC-seq, or Histone Modifications, reflecting chromatin accessibility. Also, additional features such as nucleotide composition, DNA shape, or sequence conservation can be incorporated into the predictions. Including these additional data sets and information improved the TF binding predictions considerably ^5– 12^. A non-exhaustive overview is provided in 13. While PWM based models are still the most common means to assess the likelihood of a TF binding to genomic sequences, more elaborate approaches that capture nucleotide dependencies, have been successfully used as well ^14, 15^. SLIM-models ^16^ are an example for such approaches. In contrast to other methods, nucleotide dependency profiles inferred by SLIM models can be visually interpreted. Recently, deep learning methods have been used to learn TF binding specificities *de novo* from large scale data sets comprising not only ChIP-seq but also Selex and protein binding microarray (PBM) data ^17^.

The *ENCODE-DREAM in vivo Transcription Factor binding site prediction challenge* ^18^ aims to systematically compare various approaches on TFBS prediction in a controlled setup, with the additional complexity of applying the classifiers on the tissues/cell types that were not used for model training. The *challenge* organizers provide TF-ChIP seq data for 31 TFs, accompanied with RNA-seq and DNase1-seq data in 12 different tissues. Using labels deduced from the TF-ChIP-seq data, predictive models for TF binding should be learned and then applied to a set of held-out chromosomes on an unseen tissue. Predictions are computed in bins, covering the entire target chromosomes. The main *challenge* paper will provide a detailed explanation of the *challenge* setup and a comparison across all competing methods. This article is a companion paper to the main *ENCODE-DREAM Challenge* paper, in which we describe our contribution to the *challenge*, delineate the motivation for our work and provide an independent evaluation of our ideas to achieve generalizability across tissues.

We developed an ensemble learning approach using random forest (RF) classifiers, extending the work of Liu *et al*. ^12^. Tissue-specific cofactor information was shown to be relevant to accurately model TF binding ^12, 19^. Thus, we designed our approach to aggregate tissue-specific cofactor data, via an ensemble step, into a generalizable model. Briefly, we compute TF affinities with TRAP ^20^ for 557 PWMs in DNase-hypersensitive sites (DHSs) identified with JAMM ^21^. TF affinities computed by TRAP are inferred from a biophysical model. In contrast to a simple binary classification, e.g. FIMO ^22^, these scores can capture low affinity binding sites, which were shown to be biologically relevant ^23, 24^. Here, we show that our ensemble models generalize well between tissues and that they exhibit better classification performance than tissue-specific RF classifiers. Furthermore, we illustrate that only a small subset of TF features is sufficient to predict tissue-specific TFBSs and also show that these TFs are often known co-factors/interaction partners of the target TF.

## Methods

### Data

Within the scope of the *challenge* participants were provided with ChIP-seq data for 31 TFs, as well as DNase1-seq and gene expression obtained from RNA-seq data for 13 tissues. From the available 31 TFs, 12 were used to assess the model performance in the final round of the *challenge*. As we focus in this article on the generalizability of our models, we use only those TFs that are linked to multiple training tissues. Thus, we consider the TFs listed in Table 1 for model training and general evaluation experiments. Furthermore, we use eight TFs, as provided in Table 2, to evaluate the performance of our models on unseen test data. The *challenge* required that the predictions are made in bins of size 200 *bp*, shifted by 50 *bp* each, spanning the whole genome. Except for the held-out chromosomes 1, 8, and 21, all chromosomes are used for model training. We refer to the *challenge* website for a detailed overview on the provided data ^18^. Note that we exclude sites labelled as ambiguously bound from this study.

**Table 1.** Number of bins labeled as bound per transcription factor (TF) and tissue, deduced from TF ChIP-seq data.

TF	Number of bins labelled as bound per tissue
ATF7	272,2234 (GM12878), 218,239 (HepG2), 345,775 (K562)
CREB1	164,968 (GM12878), 103,752 (H1-hESC), 178,080 (HepG2), 98,554 (K562)
CTCF	179,672 (A549), 271,097 (H1-hESC), 206,336 (HeLa-S3), 208,868 (HepG2), 215,238 (K562), 305,547 (MCF-7)
E2F1	93,117 (GM12878), 55,391 (HeLa-S3)
EGR1	72,595 (GM12878), 52,733 (H1-hESC), 175,994 (HCT116), 58,793 (MCF-7)
EP300	126,409 (GM12878), 69,247 (H1-hESC), 157,629 (HeLa-S3), 168,173 (HepG2), 137,369 (K562)
GABPA	26,467 (GM12878), 51,666(H1-hESC), 31,202 (HeLa-S3), 60,552 (HepG2), 109,423 (MCF-7), 78,403 (SK-N-SH)
JUND	203,665 (HCT116), 179,999 (HeLa-S3), 183,558 (HepG2), 193,814 (K562), 92,905 (MCF-7), 222,013 (SK-N-SH)
MAFK	34,054 (GM12878), 97,659 (H1-hESC), 62,124 (HeLA-S3), 291,337 (HepG2), 201,157 (IMR90)
MAX	301,615 (A549), 98,327 (GM12878), 224,379 (H1-hESC),
	321,501 (HCT116), 211,590 (HeLa-S3), 317,579 (HepG2), 318,318 (K562), 250,775 (SK-N-SH)
MYC	57,512 (A549), 91,325 (HeLa-S3), 183,627 (K562), 151,748 (MCF-7)
REST	71,251 (H1-hESC), 47,654 (HeLa-S3), 67,453 (HepG2), 59,640 (MCF-7), 48,946 (Panc1), 94,082 (SK-N-SH)
RFX5	161,689 (GM12878), 22,948 (HeLa-S3), 54,961 (MCF-7)
SRF	21,495 (GM12878), 40,201 (H1-hESC), 176,158 (HCT116), 22,593 (HepG2), 18,895 (K562)
TAF1	87,109 (GM12878), 185,027 (H1-hESC), 93,824 (HeLa-S3), 110,385 (K562), 83,276 (SK-N-SH)
TCF12	51,798 (GM12878), 104,834 (H1-hESC), 82,102 (MCF-7)
TCF7L2	100,926 (HCT116), 165,264 (HeLa-S3), 143,025 (Panc1)
TEAD4	66,198 (A549), 103,483 (H1-hESC), 174,716 (HCT116), 125,917 (HepG2), 186,759 (K562)
YY1	136,621(GM12878), 195,489 (H1-hESC), 63,293 (HCT116), 133,943 (HepG2)
ZNF143	197,385 (GM12878), 178,088 (H1-hESC), 48,154 (HeLA-S3), 103,755 (HepG2)

**Table 2.** Test data used in this article, shown per transcription factor (TF) and tissue.

TF	Tissues
CTCF	PC-3, Induced pluripotent stem cell
E2F1	K562
EGR1	liver
GABPA	liver
JUND	liver
MAX	liver
REST	liver
TAF1	liver

### Data preprocessing and feature generation

In order to obtain datasets per tissue and per TF that could be handled in terms of memory consumption and processing time, and also to cope with the large imbalance number of bound and unbound sites, we randomly sampled as many negative sites from the provided ChIP-seq *tsv* files as there were true binding sites per TF. The ChIP-seq labels contained in the balanced and down-sampled *tsv* files are used as the response for training RF models.

Throughout the course of the *challenge*, we have used two distinct ways to generate features for the RF classifiers: (1) with and (2) without considering DHSs. In none of the approaches have we used the provided RNA-seq data nor did we compute DNA shape features. Generally, we computed TF binding affinities with *TRAP* ^20^ for 557 distinct TFs using the default parameter settings. Within our workflow, we first consider all 557 TFs to determine factors that are predictive for the binding of the target TF. The position specific energy matrices (PSEMs) used in our computation are converted from position weight matrices (PWMs) obtained from JASPAR ^25^, UniPROBE ^26^, and Hocomoco ^27^. The code to perform the conversion and to run TRAP is available on GitHub.

**Figure 1.**
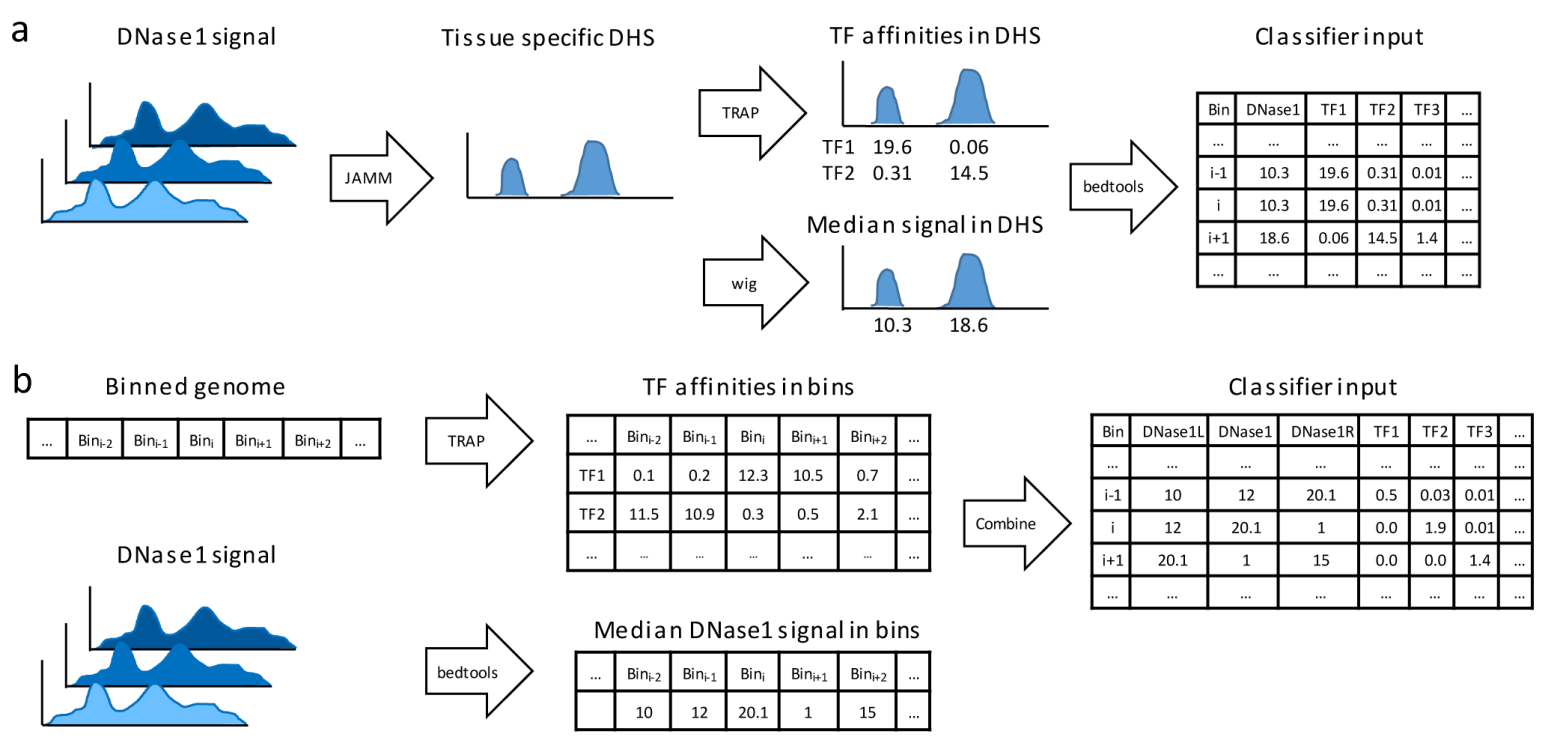
( **a**) Data pre-processing workflow using DNase1-seq Hypersensitive Sites (DHSs). Using JAMM, DHSs are called considering all available replicates for a distinct tissue. Transcription factor (TF) affinities in the identified DHSs are computed using TRAP for 557 TFs, the median signal of DHSs is assessed using bedtools. ( **b**) An alternative data pre-processing workflow without DHSs: TF affinities and median DNase1-seq signal are computed per bin.

We compared two approaches to generate features for the classifier from DNase1-seq data. In the first approach, shown in Figure 1a, we compute tissue-specific DHSs using the peak caller JAMM ^21^ (version 1.0.7.2). Specifically, we converted the provided DNase1-seq *bam* files to *bed* files using the bedtools ^28^ *bamtobed* command ( bedtools version 2.25.0). For each *bed* file, peaks are computed separately using JAMM’s standard parameters and the *–f 1* option. The individual DHS files obtained for one TF are aggregated using the bedtools *merge* command. We decided to take a less conservative approach and merge all peaks identified in individual replicates per TF to ensure that we do not miss any accessible site, all be it this may introduce false positives. Next, TF affinities are calculated in the merged DHS sites using TRAP, and the median DHS signal per peak is computed from the provided *bigwig* files. The computed data are intersected, using a *left outer join* with bedtools, with the binned genome structure required for training (using the bins contained in the *tsv* files mentioned above) and testing (using the provided *bed*-file containing all test regions).

The second approach for computing the features is depicted in Figure 1b. Here, we do not use the information on DHS sites, instead we compute TF binding affinities and the DNase1-seq signal per bin using the bin structure defined by the challenge as explained in the Data section above. We obtain the features genome-wide, without any preselection of the bins. To account for variability between both biological and technical replicates, we calculate the median DNase1-seq coverage across the replicates using the *bedtools coverage* command. Overall, the features for a single bin are composed of the TF affinities in that bin, the DNase1-seq signal in the bin itself together with its left and right neighboring bins.

### Ensemble random forest classifier

**Figure 2.**
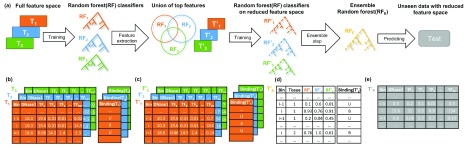
**a**) An overview of model training for a distinct transcription factor, TF, with multiple training tissues. Using the full feature matrices *T*_1_, *T*_2_, *T*_3_, depicted in ( **b**), TF and tissue-specific random forest (RF) classifiers are trained. From those RF classifiers ( *RF*_1_, *RF*_2_, *RF*_3_), we determine the union of the top 20 features from each RF. In this example, the union of top TFs is comprised of 24 TFs. Next, we design reduced tissue-specific feature matrices *T’*_1_, *T’*_2_, *T’*_3_, as shown in ( **c**) based on the union of the top TF features. Subsequently, tissue-specific RF classifiers ( *RF’*_1_, *RF’*_2_, *RF’*_3_) are trained on these reduced feature sets. The tissue-specific RF classifiers are applied to all training tissues and their predictions are aggregated to form the feature matrix *T’ _E_*, visualized in ( **d**), which is used to train an ensemble model ( *RF _E_*). At the testing phase the feature matrix *T' _p_* is fed to the trained ensemble model *RF _E_* to predict the labels for the unseen data ( **e**). Note that the column *Tissue* in **d**) is not included in the model but only shown here for illustration purposes. The feature matrices shown represent feature setup (1) using DNase1 Hypersensitive (DHS) sites.

**Figure 3.**
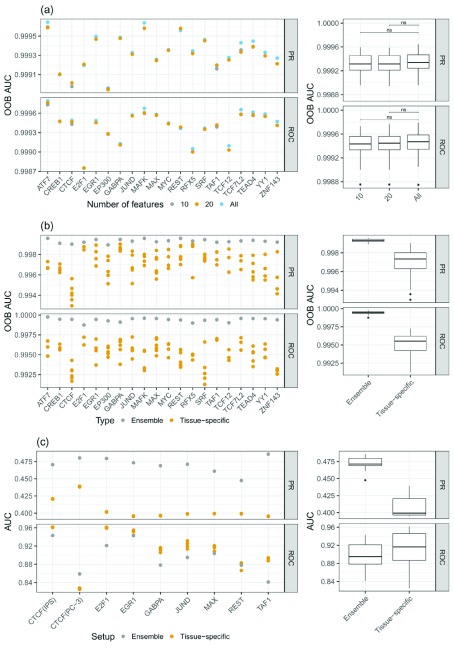
**a**) PR-AUC and ROC-AUC for different sets of features: considering *all* features, the top 10, and the top 20 features. One can see that the difference in model performance between the top 20 and *all* feature cases is only marginal. **b**) Comparison of the out of bag (OOB) error between ensemble models and tissue-specific random forest (RF) classifiers. The ensemble models show superior performance compared to the tissue-specific RF classifiers. **c**) PR-AUC and ROC-AUC computed on unseen test data for ensemble and tissue-specific RF classifiers. Due to the imbalanced nature of the test data, the ROC-AUC values are overly optimistic, as they are biased by the numerous unbound sites. However, the PR-AUC represents a more realistic view on the actual performance of the models. Note that the scale of the y-axes are different for the sub-figures.

The Random Forest models, implemented using the randomForest R-package ^29^ (version 4.6-12), are trained on either of the feature setups explained in the previous section. Training the RF models can be seen as a two-step approach that is independent from the feature setup. Throughout model training, the balance between the *bound* and *unbound* classes is maintained to avoid over-fitting of the RF classifiers and also to ensure an unbiased evaluation of model performance. For fitting the RF classifiers we used 4,500 trees, and at most 30,000 positive and negative, i.e. bound and unbound, samples. This restriction is enforced by the limitations of the *randomForest* R-package. As illustrated in Figure 2a, for a given target TF, we first learn tissue and TF specific RF classifiers using all available features from the input matrix, *T _i_* ∈ *R ^n^* ^×557^ ; *i* ∈ {1, ... ,m}, where *n* is the number of bins forming the training set, and *m* denotes the number of training tissues for the target TF:

$$RF_{i}=RandomForest(T_{i,}Binding(T_{i})),$$

where *Binding*( *T _i_*) is a vector of length *n*, holding the binding labels for the target TF in tissue *i*, and *RandomForest*(.,.) generates the RF model trained on the features and labels provided by the first and second arguments respectively. An example of the input matrix *T _i_* and the response vector *Binding*( *T _i_*) is shown in Figure 2b. In the second step, to focus only on essential regulators (c.f. Figure 3a), we shrink the feature space to the union of the top t regulators (t={10,20}) taken over all tissue and TF specific RF classifiers, $T_{i}^{\prime }$, by ranking the predictors according to their *Gini index* ( Figure 2c):

$$T_{i}^{\prime }=Subset(T_{i},\overset{m}{\underset{j=1}{\cup }}TopFeatures(RF_{j})),$$

where *TopFeatures*( *RF _j_*) denotes the top t features of *RF _j_* and *Subset*(., .) generates the reduced feature matrix based on the union of the top TFs. In the following, we refer to a training data set comprised of only one tissue as a *single tissue case* and to a training data set composed of multiple tissues as a *multi tissue case*. Considering the single tissue case, where *i* = 1 we train an RF model, $RF_{i}^{\prime }$, on the reduced feature space and use this as the final model for the respective target TF:

$$RF_{i}^{\prime }=RandomForest(T_{i}^{\prime },Binding(T_{i})).$$

In the multi-tissue scenario, we retrain tissue-specific RF models on the reduced feature space and apply them across all available training tissues:

$$T_{E}^{'}=\{prediction(RF_{i}^{'},T_{i}^{'})\},i\in \{1,\cdots ,m\},$$

where *Prediction* ( $RF_{i}^{\prime }$, $T_{i}^{\prime }$) returns the predictions made by $RF_{i}^{\prime }$ when applied on $T_{i}^{\prime }$. Thus, $T_{E}^{\prime }$ is a *n x m* matrix with values between 0 and 1, holding the predictions of the tissue specific RFs trained for the target TF on different tissues. Matrix $T_{E}^{\prime }$ is used as input for the ensemble model *RF _E_*. The ensemble model is optimised to predict the binding of the target TF based on the concatenation of predictions obtained from the training tissues. The concatenation of all binding labels is denoted by *Binding*( $T_{E}^{\prime }$) , ( Figure 2d):

$$RF_{E}=RandomForest(T_{E}^{\prime },Binding(T_{E}^{\prime })).$$

By design, the ensemble model incorporates the tissue-specific RF classifiers in a non-linear way to better generalize across all provided training tissues. An example matrix that is used to obtain predictions from an ensemble RF is shown in Figure 2e.

### Performance assessment

We assessed model performance in two different scenarios: Firstly, while fitting the RF classifiers, we measure the out-of-bag (OOB) error, which is defined as the mean prediction error for each training sample using trees that were not trained on that sample. The performance on OOB data is computed in terms of the area under the precision recall curve (PR-AUC) and the area under the receiver operator characteristic curve (ROC-AUC) using the PRROC ^30^ package. The latter contrasts false positive rate against true positive rate, while the former contrasts precision against recall. A ROC-AUC value around 0.5 suggests a random classifier. Note that there exists no random baseline for PR-AUC.

In addition to the curve based measurements, we considered the misclassification rate separately for the *Bound* and *Unbound* classes, denoting the false negative and false positive rate, respectively:

$$Bound(False\;negative\;rate)=\frac{FN}{TP+FN}\;,\;Unbound\;(False\;positive\;rate)=\frac{FP}{TN+FP}\;,$$

where *TP* denotes the bins correctly predicted as bound, *TN* denotes the bins correctly predicted as unbound, *FP* and *FN* represent bins incorrectly predicted as bound and unbound, respectively. Note that, because we use balanced data for training the RF classifiers, the OOB is computed on a balanced data set.

Secondly, we compute the aforementioned performance measurements for a subset of the test data that was used by the *challenge* organizers. As mentioned above, the test data is composed of three held-out chromosomes, which have not been used for training: 1, 8, and 21. Additionally, TF binding is predicted on an unseen tissue, i.e. a tissue that was not used for training. An overview of the test data is provided in Table 2. Note that, in contrast to the training data, the test data is not balanced, i.e. the *Unbound* class is larger than the *Bound* class. Here, we remind the reader that PR-AUC is robust against class imbalance and thus a more appropriate performance metric for the test data than ROC-AUC as well as both false positive and false negative rates. Due to memory limitation of the PRROC package we had to downsample the test data to 100,000 samples, while preserving the original *Bound* to *Unbound* ratio.

Note that, because both suggested feature setups depicted in Figure 1 are evaluated on the same gold standard (the same test data sets), their performance can be contrasted.

### Protein-protein-interaction score

By reducing the feature space of the RF models, we assumed to select TFs that are likely to interact with the target TF. To test this hypothesis systematically, we used a protein-protein-interaction score.

We obtained a customized protein-protein-interaction (PPI) probability matrix *R* as described previously ^31^, which is derived from a random walk analysis on a protein-protein-association network based on STRING ^32^ (version 9.05). An entry *R _i,j_* represents the probability that protein *i* interacts with protein *j*. Note that the probability *R _i,j_* is not symmetric by construction, i.e. *R _i,j_* ≠ *R _j,i_* . To generate a score describing how likely it is that a subset of proteins *P* contained in *R* interact with a distinct TF *t*, guided by the feature importance the RF models provide, we define the PPI score *S _t,P_* as

$$S_{t,P}=-log\text{(}\frac{\sum_{p\in P}\left((R_{p,t}+R_{t,p})\times GI(p)\right)}{\text{2|}P\text{|}}\text{),}\;(1)$$

where *GI* ( *p*) denotes the Gini index values of *p* obtained from the RF model corresponding to *t*. Thus, the smaller the value of *S _t,P_* the more likely it is that the regulators in *P* interact with TF *t*.

## Results

In this section, we first show that shrinking the feature space to those TFs essential for training does not affect model accuracy. Next, we demonstrate the benefits of the ensemble learning and how its accuracy is depending on the number of training tissues. We further investigate the top selected TFs by the RF models and find known interaction partners that possess high PPI scores. Finally, we compare the two feature design schemes, described in the *Methods* section, and explore their influences on model performance. If not stated otherwise, all figures presented in the following are based on annotation setup (1), focusing on DHSs.

### Reducing the feature space to a small subset does not affect classification performance

Because having a sparse feature space simplifies model interpretation, we reduce the feature space to contain only a few essential features. As explained above, we determined sets of top features using the Gini index, resulting in TF and tissue-specific sets containing either the top 10 or top 20 features. As shown in Figure 3a (Supplementary Figure 3a) ^33^ the difference in OOB error between the feature set comprised of the top 10 or top 20 features and the full feature space is not significant. Interestingly, on test data we see a slight increase in model performance for the reduced feature space models compared to the full model. This is most likely owing to a better generalizability of the reduced feature space (Supplementary Figure 1) ^34^. Due to the performance gain on test data, as well as a substantial improvement in interpretability of the models and in runtime, we decided to use a reduced feature space that consists of the top 20 features per model.

Our results indicate that the most important feature across all TFs is the DNase1-seq signal within the DHSs for feature setup (1). Similarly, in feature setup (2), the DNase1-seq signal within the bins is found to be more important than the TF features ( Figure 7).

### Ensemble learning improves model accuracy

According to the OOB error shown in Figure 3b (Supplementary Figure 3b) ^33^, the ensemble RF classifiers outperform the tissue-specific RFs, suggesting the ability of the ensemble model to generalize across tissues. Additionally, we assessed model performance on all test tissues, which are linked to multiple training tissues ( Figure 3c). As illustrated in Figure 3c, the PR-AUC is higher for the ensemble models than for tissue specific RFs. Due to the imbalanced nature of the test data, we observe that ROC-AUC is actually in favor of the tissue specific models. However, this is an example for an instance where ROC-AUC is not a suitable performance metric, as it is biased by the high number of negative (i.e. unbound) cases in the test data. The superior performance of the ensemble model is also illustrated by false positive and negative rates, shown in Supplementary Figure 3c ^33^.

To further demonstrate the applicability of the ensemble approach, we performed a within and across tissue comparison for ensemble and tissue specific RFs. In detail, we learned tissue specific RFs for one TF in all available training tissues as well as one ensemble model. Next, we applied each classifier on each tissue and contrasted their performance (Supplementary Figure 2) ^35^. We observe that the ensemble models perform either at least as good, or better, than the tissue specific models applied to the same tissue they were trained on. Further, while we see a decrease in the predictive power of tissue specific models applied across tissues, the performance of the ensemble model remains almost constant.

Overall, we conclude that ensemble learning is a promising approach to deal with the tissue-specificity of TF binding.

### Increasing the number of training tissues improves prediction accuracy

**Figure 4.**
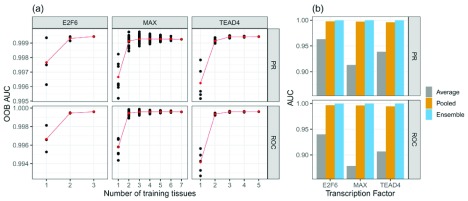
Comparison of tissue number and classifier setups for the three TFs E2F6, MAX, and TEAD4. **a**) Model performance as a function of number of tissues used for training. The OOB reduces if more tissues are included in the ensemble learning. Red dots represent the mean classification error across all tissue-specific classifiers. The black points represent individual models. **b**) Comparison between two ensemble models: averaging (takes the average of all individual RF predictions) and the RF ensemble model. In addition, one RF classifier was trained on pooled data sets comprised of training data for all available tissues for one target TF. The ensemble models perform better than the models based on aggregated data

Although the results in Figure 3b and 3c suggest that the ensemble methods perform well, it remains unclear what influence the number of training tissues would have on the performance of an RF. To elucidate this, we performed permutation experiments learning multiple RF models using all possible combinations of training tissues that are available for a distinct TF. As this is a computationally demanding task, we performed it for only three, arbitrarily selected, TFs: MAX, TEAD4, and E2F6. Figure 4a (Supplementary Figure 4a) ^36^ illustrates that the performance on OOB data improves when the number of training tissues increases. Hence, we conclude that the ability of an ensemble RF to generalize across tissues improves with larger number of training tissues.

However, it remains to be shown whether the improved accuracy obtained from the ensemble RF classifiers was in fact because of the ensemble learning. To test this, we designed two additional learning setups. Firstly, we aggregated all tissue-specific data sets into one. In other words, we pooled the training data for one TF across all available tissues into one data set. Then, we used this pooled data set to train a new RF model. Secondly, we examined another ensemble approach, which we consider to be a baseline for our actual ensemble model. In detail, we computed the average of predictions over tissue specific models in order to obtain the final prediction. As depicted in Figure 4b the true ensemble models perform better than both tested alternatives. This shows that the ensemble technique is better suited to capture tissue-specific information than simple data aggregation approaches.

### Predictors selected by the RF classifiers are associated to the target TF

**Figure 5.**
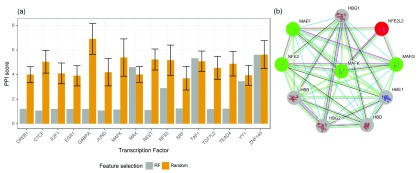
**a**) Log transformed PPI scores computed for a set of TFs. In the *Random* case, we show the mean PPI score across 100 random draws and its standard deviation. The smaller the PPI score the better. Only for three TFs ( *MAX, TAF1, ZNF143*), the randomly sampled PPI score is better than or equal to the score derived for the TFs selected by the RF classifiers. **b**) PPI network obtained from STRING centered around the TF *MAFK*, highlighting proteins that interact with *MAFK* with high confidence. Proteins colored in green were identified as important features in the RF classifiers, proteins shown in grey could not be retrieved by our model, because they are DNA-binding proteins, or we do not have a PWM for them in our set. Regulators shown in red could have been detected by the RF, but were not included in the top set of regulators.

As stated before, we hypothesized that the top predictors selected by the RF classifiers represent regulators that either exist in protein complexes with the target TF via direct or indirect binding, or bind directly to DNA in close proximity to the target TF. To investigate this hypothesis, we computed a PPI score *S _t,P_* (see *Methods*) for the selected predictors *P* per TF *t* and compared it against scores computed for randomly sampled sets of TFs (based on 100 randomly drawn TF subsets). The PPI score *S _t,P_* for TF *t* is small, if *t* is likely to interact with the factors included in the selected predictor set *P*. In contrast, the score is high if *t* is not likely to be interacting with the factors in *P*. As shown in Figure 5a, except for three TFs (MAX, TAF1, ZNF143), the PPI score of the TFs selected by the RF is better (i.e. smaller) than the scores for the randomly selected set. This indicates that the RF classifiers select features representing regulators that are more likely to be interacting with the target TF, either directly or with indirect contacts.

Figure 5b provides an example of a PPI network focused on the TF *MAFK*. The network was obtained from the STRING database ^32^, using the settings *highest confidence* and *no more than 10 interactors* to show. The top features selected by the RF classifiers contain all known regulatory proteins in this network, except for *NFE2L2*, shown in red. Among these TFs are *MAFK* itself, *MAFF*, *MAFG* and *NFE2* (highlighted in green). The strong interactions among the small *MAF* proteins ^37^ as well as the dimerization of those with *NFE2* ^38^ have been reported in the literature before.

Interaction partners shown in grey cannot be identified by our approach as either these are proteins without regulatory functions or we do not have a PWM available for them.

### Feature design influences the FP and FN predictions

**Figure 6.**
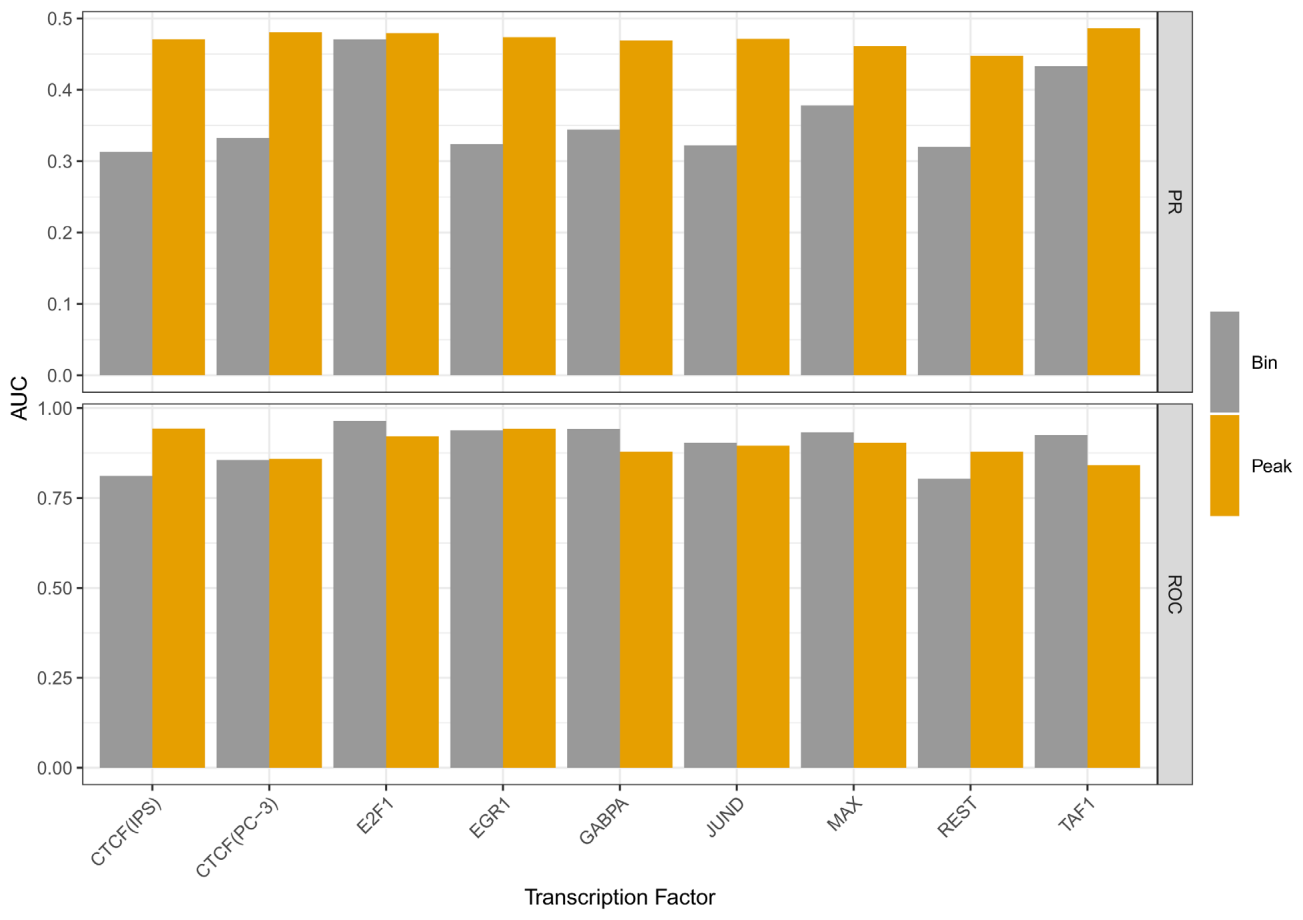
Comparison of PR-AUC and ROC-AUC for both feature setups computed on test data. In terms of PR-AUC, the peak based models clearly perform better than the bin based models. In terms of ROC-AUC it is less clear, however, as the test data is highly unbalanced, ROC-AUC is less reliable than PR-AUC.

In the conference round of the *challenge*, we were using feature setup (1), which is based on DNase1 Hypersensitive Sites (DHSs), while in the final round, we switched to design (2), which is purely based on bins. This transition had a strong effect on our performance assessed by the *challenge* organizers. While we improved the recall of our predictions by switching from (1) to (2), the precision decreased. This is reflected by the PR-AUC shown in Figure 6. Due to the unbalanced nature of the test data, which was used for this evaluation, the ROC-AUC values are less conclusive. In Supplementary Figure 5 ^39^, we show the misclassification rates for the *Bound* and *Unbound* classes depending on the two feature designs. As suggested by the PR-AUC, the bin based models (2) outperform the peak based models in the *Bound* case, whereas the peak based models show superior performance in the *Unbound* case. At the same time, bin based models perform poorly in the *Unbound* case, which is probably driven by the strong dependence of the RF classifiers on the DNase1-seq signal. In contrast to that, models based on DHSs perform well in the *Unbound* case, because the search space for TFBSs is limited to only DHSs. This increases the precision of the predictions, but at the same time lowers the recall, which is reflected by the high misclassification rate in the *Bound* case.

**Figure 7.**
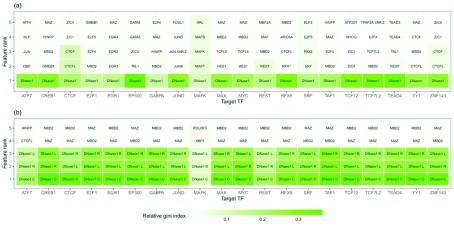
Top 5 features obtained from the average importance ranking of all tissue-specific classifiers for a given target TF shown on the x- axis for the peak setup ( **a**) and the bin setup ( **b**). Features related to DNase1 are the dominant ones.

In conclusion, according to PR-AUC and the individual error metrics, the peak based approach is the better choice.

## Discussion and conclusion

Here, we introduced an RF based ensemble learning approach to predict TFBS *in vivo*. In this article, we did not compare our approach to competitors in the *challenge*, as this is done in the main *challenge* paper. Here, we show the benefits of ensemble learning in a multi-tissue setting and that modelling cofactors is beneficial for the classification.

We show on both test and training data that the ensemble strategy is able to generalize better across tissues, than models trained on only a single tissue ( Figure 3). Also the accuracy of the ensemble classifiers increases with an increasing number of available training tissues ( Figure 4a). We also illustrate that just using all available training data to learn one RF does not provide as accurate results as an ensemble model ( Figure 4b). In this study, we decided to use RF classifiers, because they lead to accurate classification results using non-linear predictions in a reasonable time. Alternative classification approaches, such as logistic regression, or support-vector-machines could have been used too.

RF classifiers have also been proposed recently, independent from the *challenge* ^12^, as an adequate method to predict TF binding. Although the authors of 12 perform cross cell-type predictions, i.e. they predict TF binding in a tissue where the RF was not trained on, they do not use ensemble models as proposed here. However, they did show that it is beneficial for the predictions of a distinct target TF to consider further TFs as predictors, in addition to the target TF itself. This is in agreement with our findings. As shown in Figure 3a, a small subset of features is sufficient to reach similar classification performance as the full feature space. We found that most of these selected TFs are known interaction partners of the target TF, see Figure 5. This is also supported by a recent study illustrating that most TFs bind in dense clusters around genes suggesting a widespread interaction among them ^40^.

Only for three TFs, we could not find that the predicted TFs lead to a better PPI score than a randomly chosen set. We note that for two of those three, *TAF1* and *MAX*, the performance of the ensemble RF classifiers improved only marginally, or not at all, compared to the tissue-specific classifiers. This suggests that our model does not account for the true interaction partners of those TFs. Indeed, an inspection of the STRING database for *TAF1* revealed that only *TAF1* itself and *TBP* are among the top 20 regulators, which are included in our PWM collection. For the remaining interaction partners, mostly TFs of the *TAF* family, no binding motif is available in the public repositories, thus they are not included in our PWM collection and can therefore not be used by the RF classifiers. Similarly, for *MAX*, only 5 out of 20 high confidence interaction partners are included in our PWM collection. Specifically, no PWM is available for 6 TFs interacting with MAX, while the remaining interacting proteins are not categorized as TFs. Overall, our approach benefits from data availability ( Figure 4a). If there are only a few TFs available in our PWM collection, it will be harder to model the co-factor binding behavior of a TF across tissues adequately. Also, the more diverse the co-factor landscape of a TF is between the tissues, the harder it will be to learn a general model. Another crucial aspect with respect to that is the quality of the PWM. During the *challenge*, we realized that the selection of PWMs is crucial for model performance and it is required to compare PWMs obtained from different sources to make sure that one uses the one with highest information content. Nevertheless, instead of using a more recent method to model TF-motifs, we stick to the use of PWMs because they are (1) the most common way to describe the sequence specificity of TFs (2) they are available for a large number of TFs, and (3) they can be interpreted easily.

Switching the feature setup for the RF classifiers from (1) DHS-based to (2) bin-based showed that DHS sites are indispensable to the accurate TFBS predictions ( Figure 6). Using only bins, without DHS information, we could improve the recall of TFBS predictions, but only at the cost of poor precision at the same time. The explanation for this behavior is a difference in size of the genomic search space between both feature setups. The bin based models have a low misclassification rate in the *Bound* case, because they do consider the whole genome without neglecting any sites beforehand, thus improving recall. However, our observations suggest that considering only the raw signal does not sufficiently correct for false positive sites, as opposed to use DHSs, which yield an improved misclassification rate in the *Unbound* case compared to the raw signal. It might be possible to overcome the strong biases of the DHS- and the bin-based models, for instance through training yet another ensemble classifier using the predictions of the DHS- and the bin-based models as input. Depending on the application, the model could be optimized for Precision, Recall, or a joint metric like PR-AUC. 

In general, both training and evaluating TFBS prediction methods is challenging due to the class imbalance, i.e. there are many more *Unbound* (negative) than *Bound* (positive) binding sites in the genome. This requires both (a) training approaches that avoid over-fitting for one of the two classes and (b) evaluation strategies accounting for this issue. Here, we assess performance in terms of PR-AUC, ROC-AUC as well as misclassification rates separately for both positive and negative classes to deal with potential biases caused by the dominant *Unbound* case.

We note that our current investigation is not meant to construct a genome-wide classifier in which the unbound case is the most abundant. To achieve that, the highly unbalanced training data situation would need to be taken into account, for instance in the loss function of the classifier. Aside from the technical aspects, we show that modelling cofactors is helpful to predict TFBS and that ensemble learning is a promising technique to generalize information across tissues.

## Data availability

The raw data used in this study is available online at Synapse after registration and signing of a data usage policy: https://www.synapse.org/#!Synapse:syn6112317.

### Extended data

Within the Figshare repository, we provide five additional figures. Links and a brief description of the figures are provided below.

Supplementary Figure 1 ( https://doi.org/10.6084/m9.figshare.9361451.v3)

PR-AUC and ROC-AUC for different sets of features: considering *all* features, the top 10, and the top 20 features on several test tissues. One can see that there is a slight advantage for the top20 and top10 model over the full model in these scenarios. The performance is shown for individual tissues in ( **a**) and separately for the size of the feature matrices in ( **b**).

Supplementary Figure 2 ( https://doi.org/10.6084/m9.figshare.9363494.v1)

Within and cross tissue comparisons for ensemble and tissue specific RFs. Model performance is assessed in terms of ( **a**) ROC-AUC and ( **b**) PR-AUC.

Supplementary Figure 3( https://doi.org/10.6084/m9.figshare.9364268.v1)

**a**) Classification error for the *Bound* and *Unbound* classes for different sets of features: considering *all* features, the top 10, and the top 20 features. One can see that the difference in model performance between the top 20 and *all* feature cases is only marginal. **b**) Comparison of the out of bag (OOB) error between ensemble models and tissue-specific random forest (RF) classifiers. Especially in the *Unbound* case, the ensemble models show superior performance compared to the tissue-specific RF classifiers. **c**) Misclassification rate computed on unseen test data for ensemble and tissue-specific RF classifiers. As in **b**) we see that the ensemble models generally outperform the tissue-specific ones. Note that the scale of the y-axis is different for the *Bound* and *Unbound* classes in ( **a**) and ( **b**).

Supplementary Figure 4 ( https://doi.org/10.6084/m9.figshare.9366923.v1)

**a**) Relation of the OOB error for three TFs (E2F6, MAX, and TEAD4) to the number of tissues used for training. The OOB reduces if more tissues are included in the ensemble learning. Red dots represent the mean classification error across all tissue-specific classifiers. Individual models are represented by the black points. **b**) Comparison between true ensemble models for E2F6, MAX, and TEAD4 and RF classifiers trained on pooled data sets comprised of training data for all available tissues. The ensemble models perform better than the models based on aggregated data.

Supplementary Figure 5( https://doi.org/10.6084/m9.figshare.9367895.v1)

Comparison of misclassification rate depending on the feature design computed on test data.

### Software availability

Code generated as part of this analysis is available on GitHub: https://github.com/SchulzLab/TFAnalysis

Archived code at the time of publication: http://doi.org/10.5281/zenodo.1409697 ^41^

License: MIT
