# Supplementary material for: Predicting transcription factor binding using ensemble random forest models
Source: F1000Res. 2019 Sep 2;7:1603. Originally published 2018 Oct 4. [Version 2] doi: 10.12688/f1000research.16200.2 (PMC6823902; doi:10.12688/f1000research.16200.2)
Supplement: Peer review history

# Peer review: Reviewer response for version 2

**DOI:** 10.5256/f1000research.22091.r53341

**Published:** 2019-10-30

**Authors:** Gary D Stormo

**Affiliations:** 1 Department of Genetics and Edison Family Center for Genome Sciences and Systems Biology, Washington University School of Medicine, St. Louis, MO, USA

The authors have adequately addressed my concerns and questions and I think the revised article is acceptable.

I confirm that I have read this submission and believe that I have an appropriate level of expertise to confirm that it is of an acceptable scientific standard.

# Peer review: Reviewer response for version 2

**DOI:** 10.5256/f1000research.22091.r53342

**Published:** 2019-09-10

**Authors:** Jan Grau

**Affiliations:** 1 Institute of Computer Science, Martin Luther University of Halle-Wittenberg (MLU), Halle, Germany

The authors have carefully addressed all of my previous concerns.

I confirm that I have read this submission and believe that I have an appropriate level of expertise to confirm that it is of an acceptable scientific standard.

# Peer review: Reviewer response for version 1

**DOI:** 10.5256/f1000research.17691.r42084

**Published:** 2019-01-07

**Authors:** Gary D Stormo

**Affiliations:** 1 Department of Genetics and Edison Family Center for Genome Sciences and Systems Biology, Washington University School of Medicine, St. Louis, MO, USA

This paper reports the results of this group's entry into the ENCODE-DREAM challenge. The task of the challenge was to learn a model for binding of a target TF based on ChIP-seq data and DHS data from different cell types, and to predict binding on held-out data. They focused on a subset of 12 TFs. There were two types of held-out data, three chromosomes from the same cell types as the training data, and also data from different cell types not used in training. Results are reported as classification errors independently for bound and unbound sites.

 This group did not try to learn a model (such as PWM) for the target TF, rather they used existing PWM models, available in databases, for the target TF as well as for 556 other TFs (so 557 in total; when more than one PWM was available for a TF they used the one with the highest information content). They employed a random forest (RF) approach for learning the model, and they compared variations on how the training was performed. 

 There isn't yet a summary publication of the results of the challenge, so at this time we do not know how this approach compares to others. But there are some results reported that are interesting to know regardless of the ranking of this approach.

 One variation they tested was training using all of the features (a DHS score and all of the PWM scores) versus only subsets, and ranking the features to see which are most important. They found that using only the top 20 features was essentially as good as all of them, whereas the top 10 was not. Not surprisingly, the DHS score is the most important feature. They don't state it, but I assume that the PWM for the target TF is the next most important. Is that right? It is also reassuring that the set of other TFs that rank highest in importance are enriched for TFs previously shown to interact with the target TF, indicating that their models are learning something about the coordinated regulation by multiple TFs.

 They also compared prediction accuracy on models trained on individual tissue type data, versus a model trained on all of the tissue data merged together, versus an ensemble model obtained from all of the tissue types, with each treated independently. The ensemble models performed significantly better than the others (although I would like to see a separation of results on the different types of test data - see comments below). And the models improve with additional tissue types, although for most TFs the improvement is marginal beyond three.

 Comments and suggestions:

 1. Their reporting of results is less informative than it could be. For example, instead of just reporting a classification error for each class (bound and unbound) they could show ROC or PRC curves that indicate those errors for a range of thresholds. Is the reason they don't do that because their program simply returns a binary result, bound/unbound, rather than a probability (or some quantitative score) of being bound? The results as reported highlight the intrinsic tradeoff between false positive and false negative predictions because they vary rather dramatically between different test sets, but don't provide any guidance of how one might balance the two to obtain "optimal" predictions (where optimal may depend on the usage).

 2. In Figure 3c they show results on the two types of held-out data, from left out chromosomes from the same tissues as the training data and from data from different tissues. I would like to see those two types of test data reported separately. I can easily imagine that testing on left out chromosomes from the same tissue would provide better predictions, because the same set of additional TFs are utilized within the same tissue, but that on different tissues that might not be the case and that the ensemble approach might be especially useful.

 3. I'm a little confused about the differences in the two training methods shown in Figure 1, and I think some clarification is needed. 1a is clear enough, they are just using genomic regions under DHS peaks (in a given tissue), and the training involves those that are bound by the TF (in that same tissue) and those that are not. But in 1b, is the whole genome binned (and what are bin sizes, I didn't see that stated)? And then is the training on that whole genome, so that the unbound training data enormously larger than the bound data (in fact the vast majority of the genome is not under DHS peaks so its relevance isn't clear). And then when testing the models obtained from the binned training, do they make predictions on the whole genome, or only on the DHS regions? They report that training on binned data was better, but it isn't clear to me is the assessments were the same (such as testing on the whole genome versus under the DHS peaks) which may make a difference.

 4. The word "inadmissible" occurs twice, once in the Introduction and once in the Discussion. It doesn't seem to be the right word in either case, in fact based on the context I think it is opposite of what they mean. For example, the first occurrence is "(TFs) are inadmissible to maintain and establish cellular identity....". I think "essential" or "required" are more appropriate.

I confirm that I have read this submission and believe that I have an appropriate level of expertise to confirm that it is of an acceptable scientific standard, however I have significant reservations, as outlined above.

# Response

**Published:** 2019-07-27

**Authors:** Fatemeh Behjati Ardakani

1.Their reporting of results is less informative than it could be. For example, instead of just reporting a classification error for each class (bound and unbound) they could show ROC or PRC curves that indicate those errors for a range of thresholds. Is the reason they don't do that because their program simply returns a binary result, bound/unbound, rather than a probability (or some quantitative score) of being bound? 

 We agree with the reviewer that ROC and PR curves are meaningful error measures. We did not choose those initially as we believed that the misclassification rate is a more intuitive measure. Our models do allow us to compute ROC and PR curves. In the revised version of the article, we report the area under the precision recall curve (AU-PR) as well as the area under the receiver operator characteristic curve (AUC-ROC). We have moved the misclassification to the Supplement.   

 The results as reported highlight the intrinsic tradeoff between false positive and false negative predictions because they vary rather dramatically between different test sets, but don't provide any guidance of how one might balance the two to obtain "optimal" predictions (where optimal may depend on the usage). 

 We thank the reviewer for pointing out to us that the two proposed setups could be combined. It is a thought that did not occur to us. One option would be to combine the predictions obtained using both feature setups in yet another ensemble RF model. The balancing could be controlled by a customized penalization of the model, such that either Precision, Recall, or both are optimized. We addressed this point in the discussion of our article.  

   

 ===================================================================================== 

 2. In Figure 3c they show results on the two types of held-out data, from left out chromosomes from the same tissues as the training data and from data from different tissues. I would like to see those two types of test data reported separately. I can easily imagine that testing on left out chromosomes from the same tissue would provide better predictions, because the same set of additional TFs are utilized within the same tissue, but that on different tissues that might not be the case and that the ensemble approach might be especially useful.  

 We thank the reviewer for this suggestion. Indeed we see that the ensemble model predicting tissue X as well as the classifier trained only on chromosomes of tissue X, perform equally well. In contrast when evaluating the classifiers on other cell types, the ensemble method performs better than any other classifier trained on only one tissue. The results are shown in Supplementary Figure 2. 

 ===================================================================================== 

 3. I'm a little confused about the differences in the two training methods shown in Figure 1, and I think some clarification is needed. 1a is clear enough, they are just using genomic regions under DHS peaks (in a given tissue), and the training involves those that are bound by the TF (in that same tissue) and those that are not. But in 1b, is the whole genome binned (and what are bin sizes, I didn't see that stated)? And then is the training on that whole genome, so that the unbound training data enormously larger than the bound data (in fact the vast majority of the genome is not under DHS peaks so its relevance isn't clear). And then when testing the models obtained from the binned training, do they make predictions on the whole genome, or only on the DHS regions? They report that training on binned data was better, but it isn't clear to me is the assessments were the same (such as testing on the whole genome versus under the DHS peaks) which may make a difference.  

 We agree with the reviewer that this is a bit unclear without more detailed information on the challenge setup itself. We have added a description on the training, test, and benchmarking data provided by the challenge organizers to the main text. As stated there, the challenge’s objective was to predict TF binding in bins of size 200bp, shifted by 50bp each.  

 Predictions are computed for all bins in chromosomes 1, 8, and 21, the remaining chromosomes are used for training. To train the models, all bound bins in training chromosomes as well as an equal number of randomly sampled unbound bins have been used. The DNase1-seq signal in these bins is what is used in the setup described in Figure 1b. We believed that using the RFs to learn an association between DNase1-seq signal and TF binding might outperform a peak-calling based method, therefore we have pursued this approach as well. 

 The models are assessed on the bin level for both setups. In Setup 1 (Fig1a), any bin not overlapping a DHS is predicted as unbound per default, bins overlapping a DHS are subjected to classification. In Setup 2 (Fig1b) each bin is classified. Thus, the setups can be compared. 

 We have improved the description of Setup 2 (Fig1b) in the main text. 

 ===================================================================================== 

 4. The word "inadmissible" occurs twice, once in the Introduction and once in the Discussion. It doesn't seem to be the right word in either case, in fact based on the context I think it is opposite of what they mean. For example, the first occurrence is "(TFs) are inadmissible to maintain and establish cellular identity....". I think "essential" or "required" are more appropriate.  

 We thank the reviewer for spotting this mistake.  We meant to say indispensable.

# Peer review: Reviewer response for version 1

**DOI:** 10.5256/f1000research.17691.r39062

**Published:** 2018-10-26

**Authors:** Jan Grau

**Affiliations:** 1 Institute of Computer Science, Martin Luther University of Halle-Wittenberg (MLU), Halle, Germany

Transcriptional regulation by transcription factors (TFs) is one of the fundamental steps of gene regulation. Hence, knowing the genome-wide binding regions of a TF is of great interest. Experimentally, those could be determined by ChIP-seq, which, however, is time-consuming and labor-intensive. Hence, computational prediction of cell type-specific, in-vivo transcription factor binding is highly demanded.

 In their manuscript "Predicting transcription factor binding using ensemble random forest models", Ardakani, Schmidt and Schulz present a novel method for this purpose, which is based on PWMs describing TF sequence preference, and DNase-seq data capturing chromatin accessibility. This method combines i) learning random forest (RF) classifiers on feature matrices for individual cell types, ii) shrinking feature sets, and iii) learning ensemble classifiers across cell types. The authors illustrate that within their method, peak-based DNase features seem to be favorable compared with bin-based aggregation of DNase-seq coverage. Furthermore, they demonstrate that the ensemble classifier indeed yields an overall improved performance compared with cell type-specific RFs.

 As this is a companion paper to the main publication describing the results of the ENCODE-DREAM challenge, I consider a direct comparison to other approaches dispensable in this case.

 In general, most of the methods are well described and conclusions are supported by the data.

 However, I have a few major and several minor comments regarding choices made by the authors (especially with regard to performance assessment) and the presentation of specific details of methods and results, as detailed in the following.

 Major comments:

 1. In sub-section "Data" of the Methods section, the authors state that they "focus on these 12 TFs in the scope of this article". However, this is contradicted by the list provided in Table 2 listing only 8 TFs. Results for the same 8 TFs are also shown in Fig. 6, whereas several of the remaining figures (Fig 3a/b, Fig 5) present results for a larger set of TFs, i.e., for TFs not listed in sub-section "Data".

 2. The third paragraph of sub-section "Data preprocessing and feature generation" of the Methods section is lacking details. How exactly are "tissue-specific DHSs" called using JAMM? What have been the inputs and input formats? Which peaks are merged and why?

 3. Results with regard to feature shrinkage (Fig. 3a) are only shown for OOB Misclassification. As I could imagine over-fitting effects to specifics of the training cell types, I considered an evaluation on the test data highly informative. For instance, I would imagine that we see a decrease in OOB performance when shrinking features to the top 20, whereas on the test data this model achieves a better generalization and, hence, misclassification rate.

 4. The authors chose to use misclassification separated by classes, which could also be described as false negative rate and false positive rate, as performance measure for the whole manuscript.

 For several reasons, I would consider curve-based measures, especially the (area under the) precision-recall curve the more appropriate measure for this application but also in the context of the ENCODE-DREAM challenge.

 First, we face a highly imbalanced classification problem, and the precision-recall curve has been shown to be highly informative in this case ^1^.

 Second, the areas under the ROC curve and precision-recall curve have also been used for performance assessment in the ENCODE-DREAM challenge and choosing the same performance measure in this paper would foster comparison of results to those of the challenge (especially since both use the same test data).

 Third, in the discussion of Fig. 6, the authors mention that one choice of DNase data works better for bound regions, while the other works better for unbound regions. Here, we face the typical trade-off between sensitivity and specificity (or false negative rate and false positive rate), where we are unable to decide for one option based on specific, contradictory combinations of the two measures. In the ROC curve, basically (1 - FN/(TP+FN)) would be plotted against FP/(TN+FP), so we would get a broader impression of classifier performance, including the specific points on the curve chosen by the authors.

 For these reasons, the area under the ROC curve and the area under the precision-recall curve should be included as performance measures into this study. As the authors illustrate in Fig. 2d, RF classifiers already output continuous scores that could be used for computing these curves. Technically, curves and AUC values could be computed, e.g., using the R packages PRROC or precrec.

 5. In sub-section "Ensemble learning improves model accuracy" of the Results section, I agree with the authors that the ensemble classifier performs better than the individual RFs. However, currently it remains unclear if this can really be attributed to "ensemble learning" or just to averaging effects. Hence, I would suggest to include a simple averaging over the predictions of individual RFs (those, for which the predictions are also input of RF_E) as a simple baseline model (in addition to the single RF learned on the pooled data).

 In addition, for MAX, the authors might also include results for the test data in addition to what is shown in Figure 4.

 Minor comments:

 6. In the Introduction, second paragraph, the authors state that "Most of these methods are based on position weight matrices (PWMs) describing the sequence preference of TFs," giving a reference to the publication of the 2016 update of the Jaspar database. While Jaspar indeed provides PWM models, I do not consider this an appropriate reference for the definition of PWMs in general. Specifically, I would suggest to cite the seminal works of Berg & von Hippel ^2^ and of Stormo ^3^ instead.

 7. In the Introduction, second paragraph, the authors state "PWMs indicate [...] which nucleotide is most likely to occur". From my perspective, this description is not fully accurate. The most likely nucleotide is also represented by consensus sequences. PWMs give a specific weight (or log-probability,...) for each of the nucleotides and not only for the most likely one.

 8. I appreciate that the authors reference our work regarding dependency models (Slim models) in the second paragraph of the introduction. However, there are several other approaches for modeling dependencies in TF binding sites. I would encourage the authors to broaden the scope of their references by including, e.g. ^4^^-^^5^.

 9. In the third paragraph of the introduction, the authors refer to "the main ENCODE-DREAM Challenge paper". I am aware that this paper has not yet been published, but encourage the authors to update their publication including a reference to that paper when available.

 10. In the second paragraph of sub-section "Data preprocessing and feature generation" of the Methods section, it is mentioned that TF binding affinities are computed for 557 distinct TFs. After reading the complete paper, I understood (hopefully correctly) that all 557 TFs are used for all RFs (before shrinking the feature space) regardless of the training TF. If my understanding is correct, the authors might consider to include an explicit statement about this fact already at this stage of the manuscript.

 11. In the first paragraph of sub-section "Ensemble random forest classifier" of the Methods section, the authors state that "the balance between the bound and unbound classes is maintained to avoid over-fitting". For me, it remains unclear how exactly this helps to avoid over-fitting. For my understanding, over-fitting typically refers to an over-adaption to specifics of the training data, which do not generalize well to other data sets, leading to a poor performance on unseen (test) data. However, the class imbalance is inherent to the problem and should be (roughly) the same for training and test cell types. Please clarify.

 12. In the first paragraph of sub-section "Ensemble random forest classifier" of the Methods section, right before the second formula, the shrunken feature space is described to be the union of top 20 regulators. However, later in the Results section, the authors also consider a case where features per RF are restricted to the top 10 ones (Fig 3a). Hence, I would suggest a generic description, here.

 13. The third formula of sub-section "Ensemble random forest classifier" of the Methods section refers to an index i, where (for my understanding), according to the previous definition, i should be in {1}, in this case. If that is indeed the case, I would suggest to replace "i" by "1" in the formula and explicitly state that this is the only index i can be.

 14. The fourth formula of sub-section "Ensemble random forest classifier" of the Methods section is partly broken. Specifically, the element sign refers to the set of indexes, which does not seem reasonable to me. I rather think this should refer to the matrix resulting from prediction(RF_i',T_i') Please fix.

 15. In Figure 2 (b), (c) and (e), the labels in the table cells are hardly legible in printout. Either increase the thickness of letters or chose a different color.

 16. For Figure 2e, it remains unclear from the caption what is shown. It seems to be the input matrix derived from test data, in analogy to the training matrices shown in Figure 2b? Is this the input of each RF? Of RF' (as features might have been shrunken)? Or of RF_E?

 17. The fifth formula of sub-section "Ensemble random forest classifier" of the Methods might profit from a bit of additional explanation. Specifically, it took me a while to understand (if I'm right) that for T_E', the outputs of all individual RFs are concatenated row-wise, while "Binding(T_E')" denotes the concatenation of training labels.

 18. In the first paragraph of sub-section "Performance assessment" of the Methods section, I wondered what the index "i" refers to. Is this the same index i as before (i.e., an index for the training cell types)? If not, what exactly is "sample i"?

 19. In sub-section "Protein-protein-interaction score" of the Methods section, I would have appreciated a bit more motivation before describing the method itself.

 20. In sub-section "Reducing the feature space to a small subset [...]" of the Results section, I would not fully agree with the authors that the difference in error between the full model and the model based on top 20 features is "marginal". I would even assume that a statistical test of the difference between the data behind the two boxplots in Fig. 3a would be significant.

 21. In sub-section "Reducing the feature space to a small subset [...]" of the Results section, I did not find the last two sentences (regarding importance of DNase-based features) to be supported by the data shown in the manuscript.

 22. In section "Data availability", the authors provide a link to the synapse page of the ENCODE-DREAM challenge. However, the data are accessible only after registration and signing a data usage policy.

 23. Typos & Grammar:

 - first paragraph of "Data preprocessing and feature generation": "down sampled" should be "down-sampled"

 - second paragraph of "Data preprocessing and feature generation": "the course of challenge" should be "the course of the challenge"

 - third paragraph of "Data preprocessing and feature generation": "data is intersected" should be "data are intersected"

 - 7th paragraph of "Discussion and conclusions": "Bound(positive)" should be "Bound (positive)"

 - Reference 15: "transcritpion" should be "transcription"

I confirm that I have read this submission and believe that I have an appropriate level of expertise to confirm that it is of an acceptable scientific standard, however I have significant reservations, as outlined above.

## References

[1] : The precision-recall plot is more informative than the ROC plot when evaluating binary classifiers on imbalanced datasets. *PLoS One*.2015;10(3) : 10.1371/journal.pone.0118432 e0118432 10.1371/journal.pone.0118432 25738806PMC4349800

[2] : Selection of DNA binding sites by regulatory proteins. *Journal of Molecular Biology*.1987;193(4) : 10.1016/0022-2836(87)90354-8 723-743 10.1016/0022-2836(87)90354-8 3612791

[3] : Use of the ‘Perceptron’ algorithm to distinguish translational initiation sites inE. coli. *Nucleic Acids Research*.1982;10(9) : 10.1093/nar/10.9.2997 2997-3011 10.1093/nar/10.9.2997 PMC3206707048259

[4] : Bayesian Markov models consistently outperform PWMs at predicting motifs in nucleotide sequences. *Nucleic Acids Research*.2016;44(13) : 10.1093/nar/gkw521 6055-6069 10.1093/nar/gkw521 PMC529127127288444

[5] : On the value of intra-motif dependencies of human insulator protein CTCF. *PLoS One*.2014;9(1) : 10.1371/journal.pone.0085629 e85629 10.1371/journal.pone.0085629 24465627PMC3899044

# Response

**Published:** 2019-07-27

**Authors:** Fatemeh Behjati Ardakani

1. In sub-section "Data" of the Methods section, the authors state that they "focus on these 12 TFs in the scope of this article". However, this is contradicted by the list provided in Table 2 listing only 8 TFs. Results for the same 8 TFs are also shown in Fig. 6, whereas several of the remaining figures (Fig 3a/b, Fig 5) present results for a larger set of TFs, i.e., for TFs not listed in sub-section "Data".  

We thank the reviewer for spotting this. There was indeed an error in Table 1 and some TFs were missing. We have corrected Table 1 to list all TFs considered in Figures 3 and 5. In Fig.6, as well as in Fig.3c, we show results on test data from the challenge, therefore there are fewer TFs than in the remaining Figures. As we only look at the multi-tissue cases, for which there are more than one training tissue per TF available, we use only a subset of the available challenge data. 

===================================================================================== 

2. The third paragraph of sub-section "Data preprocessing and feature generation" of the Methods section is lacking details. How exactly are "tissue-specific DHSs" called using JAMM? What have been the inputs and input formats? Which peaks are merged and why?  

We have clarified these points in the main text. We converted the provided DNase1-seq bam files to bed files using the bedtools bamtobed command. For each bed file, peaks are computed separately using JAMM’s standard parameters and the –f 1 option. The individual DHS files obtained for one TF are aggregated using the bedtools merge command. We decided to take a less conservative approach and merge all peaks identified in individual replicates per TF to ensure that we do not miss an accessible site, all be it this may introduce false positives. 

===================================================================================== 

3. Results with regard to feature shrinkage (Fig. 3a) are only shown for OOB Misclassification. As I could imagine over-fitting effects to specifics of the training cell types, I considered an evaluation on the test data highly informative. For instance, I would imagine that we see a decrease in OOB performance when shrinking features to the top 20, whereas on the test data this model achieves a better generalization and, hence, misclassification rate.  

We appreciate the suggestions and performed the same experiment as shown in Figure 3a using the challenge’s test data (Supplementary Figure 1). As expected, we find a slight decrease in terms of OOB performance for the top10 and top20 models compared to all features, whereas on test data we see that both the top10 and top20 models perform slightly better than models considering all features. However, we note that the differences are not significant. 

===================================================================================== 

4. The authors chose to use misclassification separated by classes, which could also be described as false negative rate and false positive rate, as performance measure for the whole manuscript.  

We have mentioned these more established names in the main manuscript. However, we decided to stick to the already used nomenclature, as we believe that it is more comprehensible in the context of the TF binding predictions. 

For several reasons, I would consider curve-based measures, especially the (area under the) precision-recall curve the more appropriate measure for this application but also in the context of the ENCODE-DREAM challenge.  

First, we face a highly imbalanced classification problem, and the precision-recall curve has been shown to be highly informative in this case 1.  

Second, the areas under the ROC curve and precision-recall curve have also been used for performance assessment in the ENCODE-DREAM challenge and choosing the same performance measure in this paper would foster comparison of results to those of the challenge (especially since both use the same test data).  

Third, in the discussion of Fig. 6, the authors mention that one choice of DNase data works better for bound regions, while the other works better for unbound regions. Here, we face the typical trade-off between sensitivity and specificity (or false negative rate and false positive rate), where we are unable to decide for one option based on specific, contradictory combinations of the two measures. In the ROC curve, basically (1 - FN/(TP+FN)) would be plotted against FP/(TN+FP), so we would get a broader impression of classifier performance, including the specific points on the curve chosen by the authors.  

For these reasons, the area under the ROC curve and the area under the precision-recall curve should be included as performance measures into this study. As the authors illustrate in Fig. 2d, RF classifiers already output continuous scores that could be used for computing these curves. Technically, curves and AUC values could be computed, e.g., using the R packages PRROC or precrec.  

We agree with the reviewer that curve based scores like PR and ROC are better to assess the performance of our models. As suggested, we used the PRROC package to compute AUC values for PR and ROC curves and use these measures throughout the article. It is worth noting that the PR values deliver a more realistic impression on model performance than ROC or the misclassification rate on the highly unbalanced test data sets, which are enriched with negative cases, i.e. unbound sites. We moved the previous figures using the misclassification rate to the Supplement. 

==================================================================================== 

5. In sub-section "Ensemble learning improves model accuracy" of the Results section, I agree with the authors that the ensemble classifier performs better than the individual RFs. However, currently it remains unclear if this can really be attributed to "ensemble learning" or just to averaging effects. Hence, I would suggest to include a simple averaging over the predictions of individual RFs (those, for which the predictions are also input of RF_E) as a simple baseline model (in addition to the single RF learned on the pooled data).  

We agree with the reviewer's comment, and as suggested, we added another model averaging over the predictions of the tissue specific RFs as a baseline for our ensemble models. As shown in Figure 4b, the averaging leads to a worse performance than simply pooling the information across all samples into one model, indicating that the ensemble step does indeed combine tissue specific information in a more sophisticated way than a simple average. 

In addition, for MAX, the authors might also include results for the test data in addition to what is shown in Figure 4.  

In the interest of clarity and homogeneity of the analysis, we refrained from performing the analysis for MAX additionally on test data. 

Minor comments:  

6. In the Introduction, second paragraph, the authors state that "Most of these methods are based on position weight matrices (PWMs) describing the sequence preference of TFs," giving a reference to the publication of the 2016 update of the Jaspar database. While Jaspar indeed provides PWM models, I do not consider this an appropriate reference for the definition of PWMs in general. Specifically, I would suggest to cite the seminal works of Berg & von Hippel 2 and of Stormo 3 instead.  

We agree with the reviewer and have changed the citation.  

==================================================================================== 

7. In the Introduction, second paragraph, the authors state "PWMs indicate [...] which nucleotide is most likely to occur". From my perspective, this description is not fully accurate. The most likely nucleotide is also represented by consensus sequences. PWMs give a specific weight (or log-probability,...) for each of the nucleotides and not only for the most likely one.  

This is true. We adapted the wording in the main text to avoid the confusion. 

===================================================================================== 

8. I appreciate that the authors reference our work regarding dependency models (Slim models) in the second paragraph of the introduction. However, there are several other approaches for modeling dependencies in TF binding sites. I would encourage the authors to broaden the scope of their references by including, e.g. 4- 5.  

We appreciate the hint and have included the suggested literature. 

===================================================================================== 

9. In the third paragraph of the introduction, the authors refer to "the main ENCODE-DREAM Challenge paper". I am aware that this paper has not yet been published, but encourage the authors to update their publication including a reference to that paper when available.  

Yes, we will cite this paper once it is available. Up to the submission of this revised version of our article, the challenge paper has not yet been published. 

===================================================================================== 

10. In the second paragraph of sub-section "Data preprocessing and feature generation" of the Methods section, it is mentioned that TF binding affinities are computed for 557 distinct TFs. After reading the complete paper, I understood (hopefully correctly) that all 557 TFs are used for all RFs (before shrinking the feature space) regardless of the training TF. If my understanding is correct, the authors might consider to include an explicit statement about this fact already at this stage of the manuscript.  

Yes, the modelling is performed exactly in that way. We have improved the wording to make this more pronounced at the specified position in the main text.  

===================================================================================== 

11. In the first paragraph of sub-section "Ensemble random forest classifier" of the Methods section, the authors state that "the balance between the bound and unbound classes is maintained to avoid over-fitting". For me, it remains unclear how exactly this helps to avoid over-fitting. For my understanding, over-fitting typically refers to an over-adaption to specifics of the training data, which do not generalize well to other data sets, leading to a poor performance on unseen (test) data. However, the class imbalance is inherent to the problem and should be (roughly) the same for training and test cell types. Please clarify.  

Yes, the term “overfitting” might have been inaccurate when class imbalance was considered. We mean the class imbalance effects on training would've been attenuated by keeping the balance between bound an unbound in our training data. The class distribution of the test data, however, would not be a problem given that the models are fairly and reliably trained. 

===================================================================================== 

12. In the first paragraph of sub-section "Ensemble random forest classifier" of the Methods section, right before the second formula, the shrunken feature space is described to be the union of top 20 regulators. However, later in the Results section, the authors also consider a case where features per RF are restricted to the top 10 ones (Fig 3a). Hence, I would suggest a generic description, here.  

We thank the reviewer to point out this inconsistency. We have replaced the numeric value by a generic description. 

===================================================================================== 

13. The third formula of sub-section "Ensemble random forest classifier" of the Methods section refers to an index i, where (for my understanding), according to the previous definition, i should be in {1}, in this case. If that is indeed the case, I would suggest to replace "i" by "1" in the formula and explicitly state that this is the only index i can be.  

The observation is correct. We adapted the text accordingly. 

===================================================================================== 

14. The fourth formula of sub-section "Ensemble random forest classifier" of the Methods section is partly broken. Specifically, the element sign refers to the set of indexes, which does not seem reasonable to me. I rather think this should refer to the matrix resulting from prediction(RF_i',T_i') Please fix.  

We thank the reviewer for spotting this mistake. We have corrected it. 

===================================================================================== 

15. In Figure 2 (b), (c) and (e), the labels in the table cells are hardly legible in printout. Either increase the thickness of letters or chose a different color.  

We have increased the font size. 

===================================================================================== 

16. For Figure 2e, it remains unclear from the caption what is shown. It seems to be the input matrix derived from test data, in analogy to the training matrices shown in Figure 2b? Is this the input of each RF? Of RF' (as features might have been shrunken)? Or of RF_E?  

Indeed, in Figure 2e the input matrix for the test instances is shown. The matrix is used as input for the individual classifiers T′1, T′2, T′3, which is the classifiers learned on the reduced feature space. We have improved the figure legend to better address this point. 

===================================================================================== 

17. The fifth formula of sub-section "Ensemble random forest classifier" of the Methods might profit from a bit of additional explanation. Specifically, it took me a while to understand (if I'm right) that for T_E', the outputs of all individual RFs are concatenated row-wise, while "Binding(T_E')" denotes the concatenation of training labels.  

We reformulated the text for better clarity. 

===================================================================================== 

18. In the first paragraph of sub-section "Performance assessment" of the Methods section, I wondered what the index "i" refers to. Is this the same index i as before (i.e., an index for the training cell types)? If not, what exactly is "sample i"?  

We have removed the index. It was not required at this point. 

===================================================================================== 

19. In sub-section "Protein-protein-interaction score" of the Methods section, I would have appreciated a bit more motivation before describing the method itself.  

We have added a sentence for motivation. 

===================================================================================== 

20. In sub-section "Reducing the feature space to a small subset [...]" of the Results section, I would not fully agree with the authors that the difference in error between the full model and the model based on top 20 features is "marginal". I would even assume that a statistical test of the difference between the data behind the two boxplots in Fig. 3a would be significant.  

We performed a statistical test on the difference of PR-AUC and ROC-AUC for both the OOB error as well as the test data (Figure 3a and Sup. Fig1, respectively).  The differences are not significant for any of those instances.  

===================================================================================== 

21. In sub-section "Reducing the feature space to a small subset [...]" of the Results section, I did not find the last two sentences (regarding importance of DNase-based features) to be supported by the data shown in the manuscript.  

We appreciate that the reviewer pointed us to the lack of evidence required for this statement. We have added another Figure (Figure 7) to the main paper illustrating the feature importance of the RFs, which supports the statement made in the section mentioned above. 

===================================================================================== 

22. In section "Data availability", the authors provide a link to the synapse page of the ENCODE-DREAM challenge. However, the data are accessible only after registration and signing a data usage policy.  

We thank the reviewer for pointing this out to us. We have added it to the main manuscript.  

===================================================================================== 

23. Typos & Grammar:  

- first paragraph of "Data preprocessing and feature generation": "down sampled" should be "down-sampled"  

- second paragraph of "Data preprocessing and feature generation": "the course of challenge" should be "the course of the challenge"  

- third paragraph of "Data preprocessing and feature generation": "data is intersected" should be "data are intersected"  

- 7th paragraph of "Discussion and conclusions": "Bound(positive)" should be "Bound (positive)"  

- Reference 15: "transcritpion" should be "transcription"  

We thank the reviewer for spotting the typos, we have corrected them.
